# Supplementary material for: A G1528C Hadha knock-in mouse model recapitulates aspects of human clinical phenotypes for long-chain 3-hydroxyacyl-CoA dehydrogenase deficiency
Source: Commun Biol. 2023 Aug 29;6:890. doi: 10.1038/s42003-023-05268-1 (PMC10465608; doi:10.1038/s42003-023-05268-1)
Supplement: Supplementary file 2 — Supplementary Information [file 42003_2023_5268_MOESM2_ESM.pdf]

a

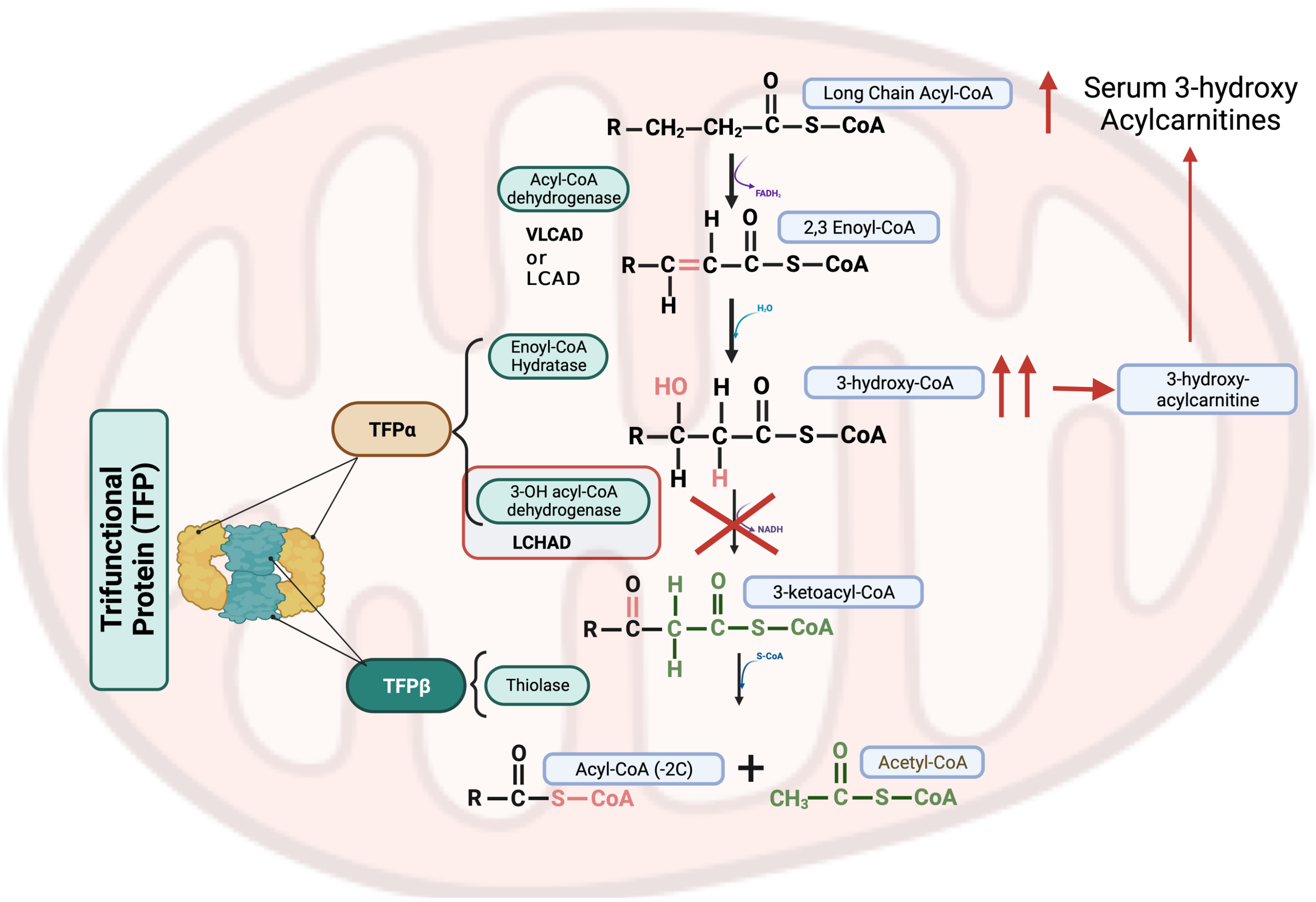

b

Human	494	VIGMHYFSPVDKMQLLEIITTEKTSKDTSASAVAVGLKQGKVIIVVK	540
Mus WT	494	VIGMHYFSPVDKMQLLEIITTDKTSKDTTASAVAVGLRQGKVIIVVK	540
Mus G1528C	494	VIGMHYFSPVDKMQLLQIITTDKTSKDTTASAVAVGLRQGKVIIVVK	540

Supplementary Figure 1: Long-Chain Fatty Acid Oxidation pathway with decreased LCHADD activity and the Conserved Human and mouse TFPα partial protein sequences surrounding the LCHADD mutation (E510Q). (a) LC-FAO in the mitochondria is mediated by very long-chain acylCoA dehydrogenase (VLCAD) and trifunctional protein (TFP). The TFPα protein contains the long-chain enoyl-CoA hydratase and long-chain 3-hydroxyacylCoA dehydrogenase activities. Humans and mice with G1528C have decreased LCHADD but relatively preserved hydratase activity. The block at LCHADD leads to accumulation of 3-OH fatty acids, and aclycarnitines that are detected in serum. (created in BioRender.com) (b) Sequences include human (NP_000173.2), mouse WT (NP_849209.1) and the mouse G1528C knock-in (theoretical mRNA translation). The position of the human 510 E>Q mutation is in red, and the silent mutation is blue. Conservative evolutionary substitutions are in gray.

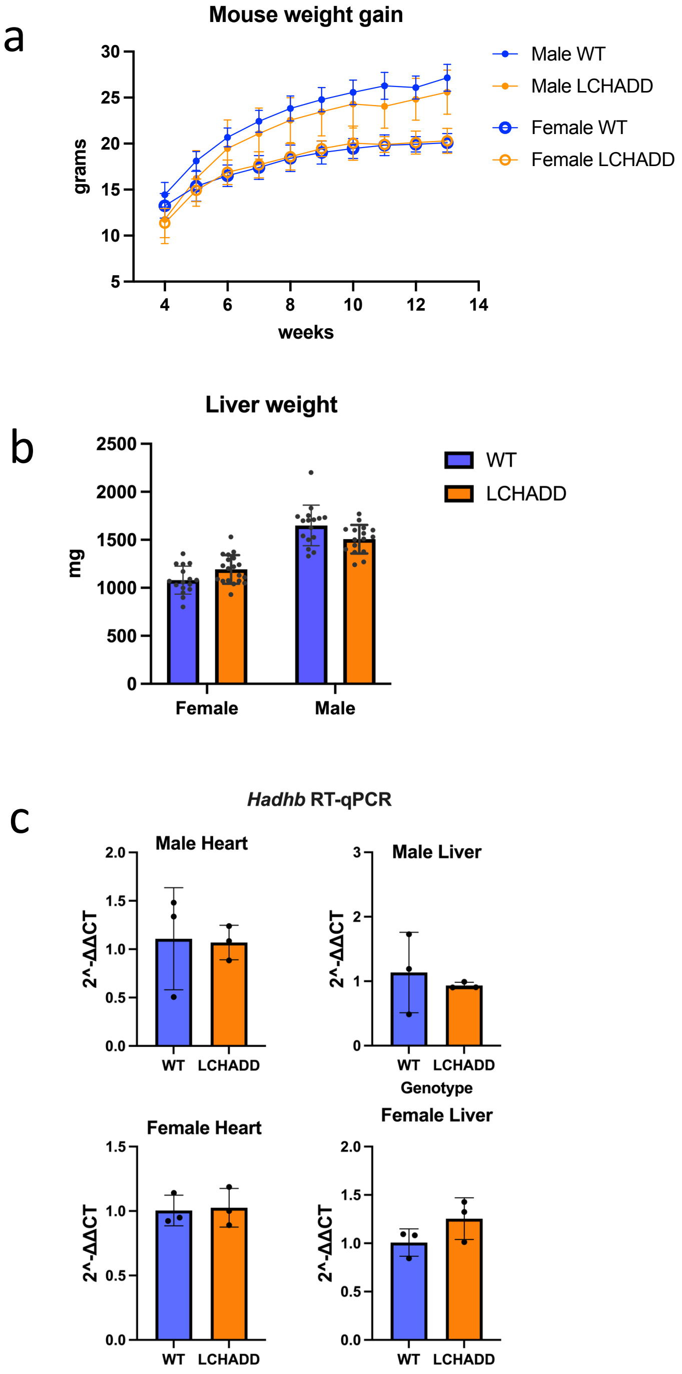

Supplementary Figure 2: Growth, liver weight and *Hadhb* gene expression. (a) Mice were weighed weekly from weeks 4-14. (WT: male n=8, female n=10; LCHAD: male n=4, female n=6). No difference was seen between genotypes of the same sex. (b) Livers of mice between 12-18 months were weighed. No differences were seen in weights. (n=16 per group). (c) *Hadhb* RT-qPCR in male and female heart and liver was not different between genotypes. Data are presented as mean $\pm$ SD.

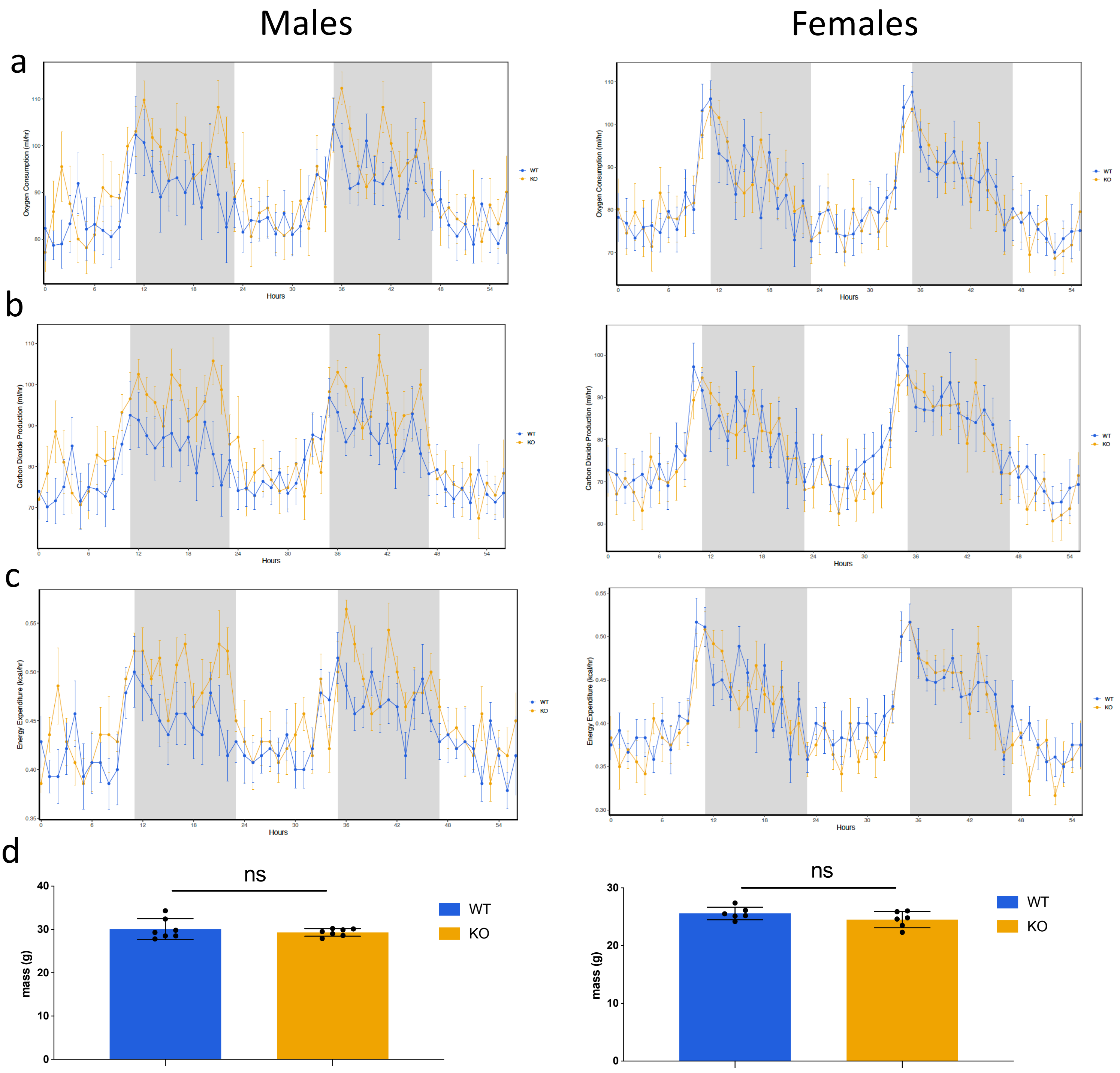

Supplementary Figure 3: Indirect calorimetry Gas exchange measures in 10 month old mice. (a) Hourly VO_2 for 48 hours in Male and Female mice was not different by genotype. (b) VCO_2 was higher during dark hours (grey shaded areas) in LCHADD males but not females. (c) Energy expenditure and (d) Body weight was not different between genotypes. WT=wildtype mice (blue) KO=LCHADD mutant mice (orange) (WT: male n=7, female n=6; LCHADD: male n=7, female n=6). Data are presented as mean ± SD; ns=no significance. Figures (a-c) from Calr analysis. Grey shaded areas for a, b and c represent time during the dark.

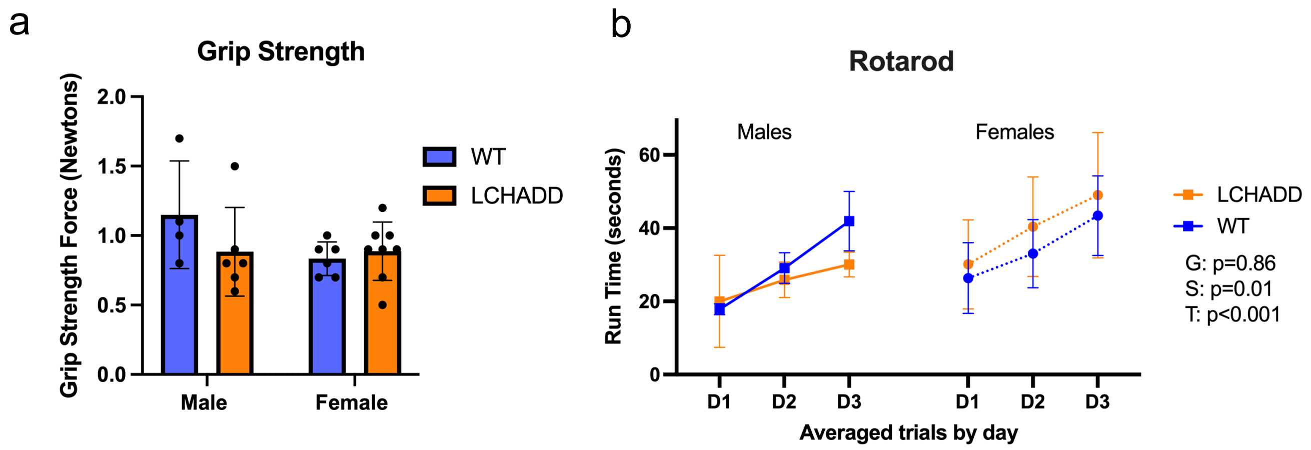

Supplementary Figure 4: Grip strength and Rotor rod testing in LCHADD and WT mice. Grip strength and Rotor Rod tests were performed in 1 year old mice. (WT males n=4, females n=4; LCHADD males n=6, female n=8 (a) There was no difference in grip strength or (b) in rotor rod run time between LCHADD and WT mice. Data are mean ± SD. (Main Effects: G=group; S=sex; T=time).

a

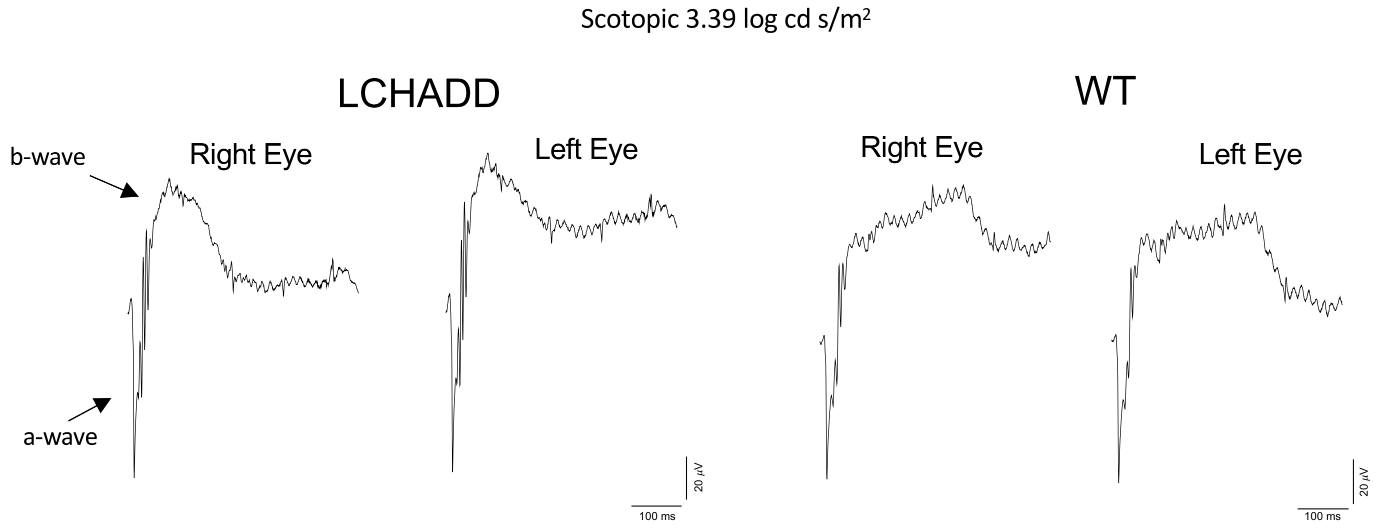

b

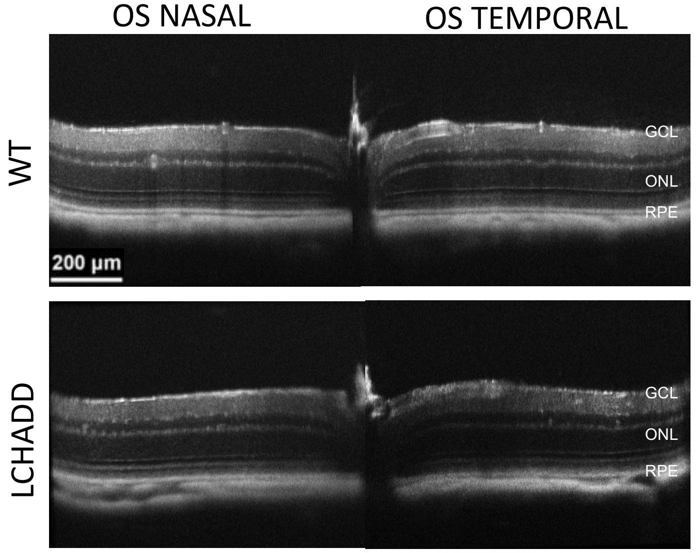

Supplementary Figure 5: Scotopic a, b ERG amplitudes and SD-OCT images. (a) Representative Scotopic a and b waveforms in LCHADD and WT mice. No difference in a and b scotopic amplitudes were seen between genotypes (b) SD-OCT images were captured in 1 year old mice (WT: n=5, LCHADD, n=8). There was no difference in SD-OCT images between genotypes. GCL-ganglion cell layer, ONL- outer nuclear layer, RPE-retinal pigment epithelium.

S6a

Heart blots

All images within a green box are from a single membrane

Key to standards

250kDa
150kDa
100kDa
75kDa
50kDa
37kDa
25kDa
20kDa
15kDa
10kDa

<u>Expected size of mature proteins</u>

TFPα = 78 kDa
TFPβ = 47kDa
VLCAD = 66 kDa
GAPDH = 34 kDa

p[Ab] = polyclonal
m[Ab] = monoclnal

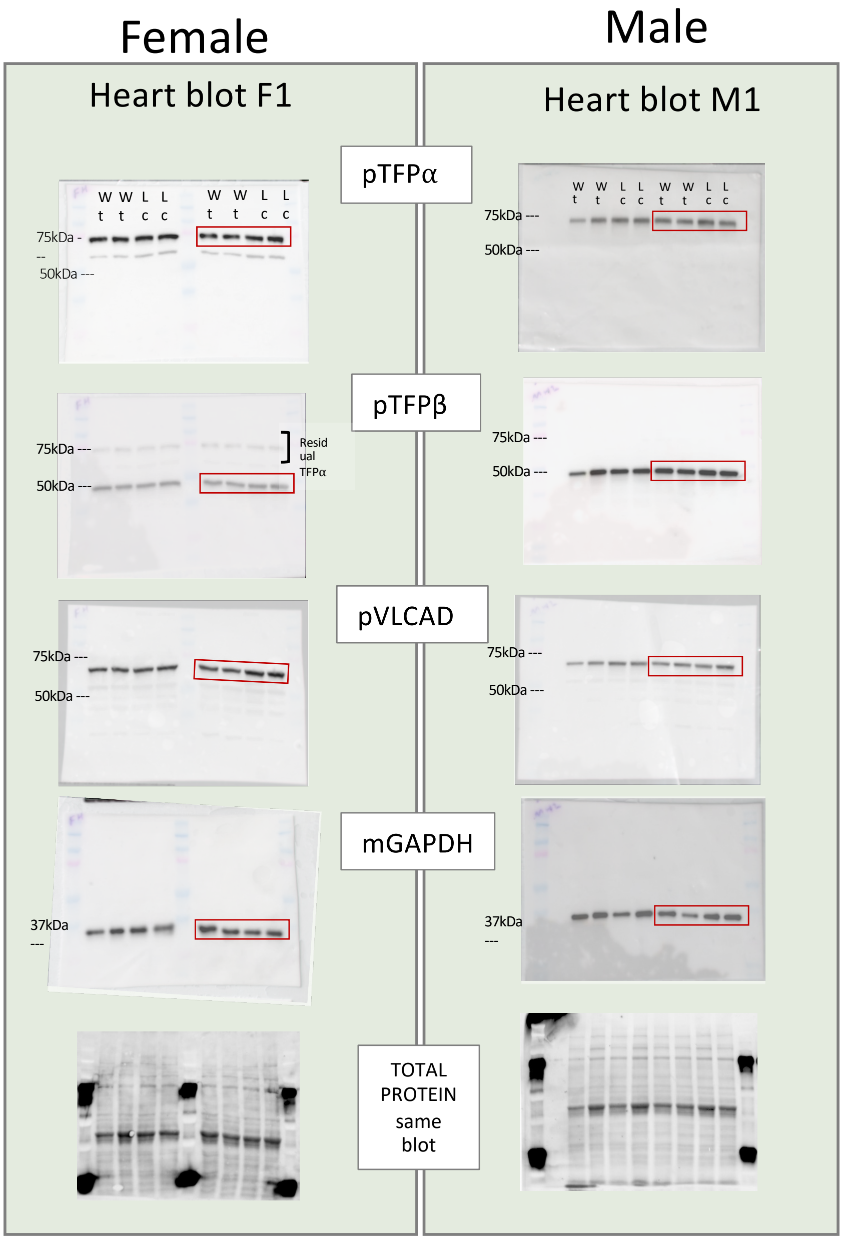

S6b

Liver blots

All images within a green box are from a single membrane

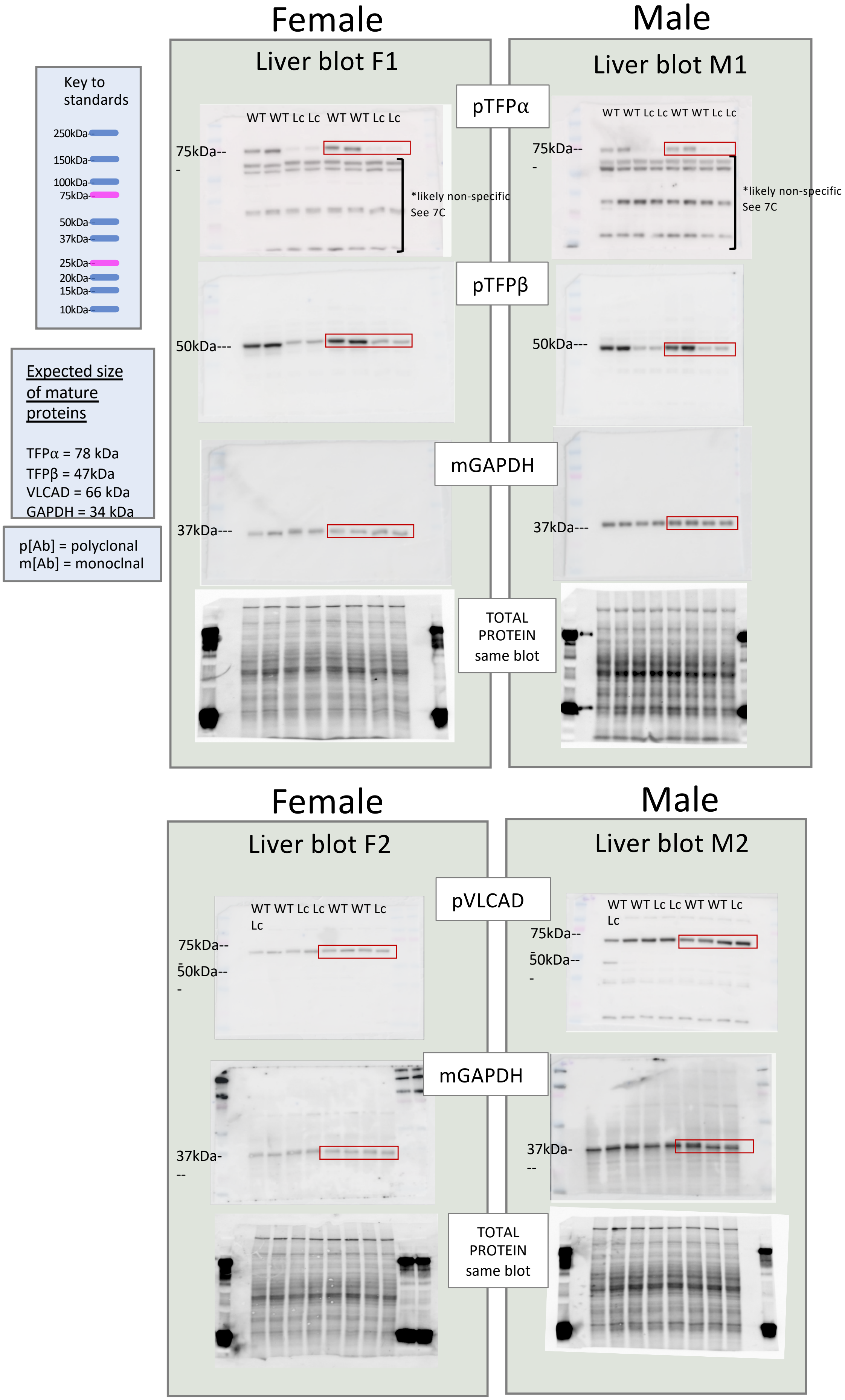

S6c Monoclonal TFPα blots

All images within a green box are from a single membrane

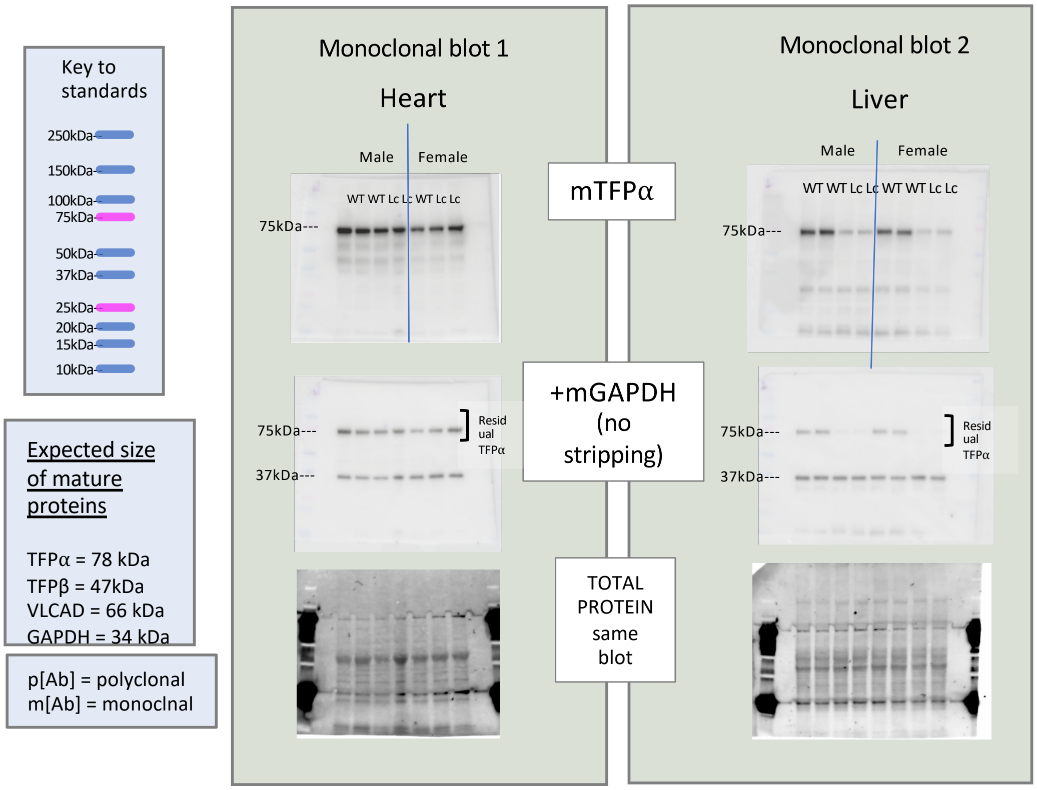

Supplementary Figure 6: Full Western blots. Homogenized heart and liver tissues from 12-15 month old WT and LCHADD mice of each sex (a, b n=4 per group; c, see figure) were run by SDS-PAGE and transferred to a membrane. The membrane was imaged for total protein, and indicated antibodies were imaged by HRP chemiluminescence. Chemiluminescent images are superimposed on the captured marker image to aid in interpretation. Images within a green box represent a single membrane. Red outlines indicate bands used in Figure 1b. Blots in a and b were stripped between probings. Wt = Wild Type, Lc = LCHADD, monoclonal and polyclonal antibodies are designated by a “m” or “p" before the target name, e.g. mAB or pAB. a) Male and female heart tissues were probed on separate blots for pTFPα, pTFPβ, pVLCAD, and mGAPDH. b) Male and Female liver tissues were probed on separate blots for pTFPα, pTFPβ, and mGAPDH. Due to stripping difficulties, additional blots were used to probe pVLCAD and mGPADH. c) Due to numerous non-specific bands using pTFPα in liver, new membranes of heart and liver were run using a monoclonal TFPα (mTFPα). All bands except the ~74kDa band fade or disappear. mGAPDH was also probed without stripping mTFPα.

Supplementary Table 1: Antibody concentrations used for western blots

Category	Target	Company	Cat #	Concentratio n
Polyclonal	HADHA	Thermo Scientific	PA527348	1:1000
Polyclonal	HADHB	Thermo Scientific	PA5117024	1:1000
Polyclonal	ACADVL	Invitrogen	PA529959	1:1000
Monoclonal	HADHA (3E9B1)	Proteintech	50-173-6347	1:1000
Monoclonal	GAPDH	Santa Cruz Biotechnology	sc-365062	1:3000
Monoclonal	GAPDH-HRP	Santa Cruz Biotechnology	sc-365062 HRP	1:2000
Secondary	Mouse TrueBlot®	Rockland Immunilogical	18-8817-30	1:2000
Secondary	Goat anti-rabbit	Thermo Scientific	31462	1:8000

Supplementary Table 2: Primer List for qPCR

Gene	Sequence (5’-3’)
Hadha FWD (mouse)	ATGGCGTCAAAGGGGATGTGGC (Exon 3)
Hadha RVS (mouse)	TGGTCGTTGGCCCAGATTTCGT (Exon 4)
Hadhb FWD (mouse)	CCCTGGGAGCTGGCTTCTCTGA (Exon 7)
Hadhb RVS (mouse)	CTCAACACCACCAGCCACGACG (Exon 8)
b-actin FWD (mouse)	CCCGGGCTGTATTCCCCTCCAT (Exon 2)
b-actin RVS (mouse)	TGGGCCTCGTCACCCACATAGG (Exon 3)
