# Supplementary material for: A G1528C Hadha knock-in mouse model recapitulates aspects of human clinical phenotypes for long-chain 3-hydroxyacyl-CoA dehydrogenase deficiency
Source: Commun Biol. 2023 Aug 29;6:890. doi: 10.1038/s42003-023-05268-1 (PMC10465608; doi:10.1038/s42003-023-05268-1)
Supplement: Supplementary file 5 — Reporting Summary [file 42003_2023_5268_MOESM5_ESM.pdf]

nature portfolio

Corresponding author(s): Melanie B Gillingham

Last updated by author(s): Aug 9, 2023

Reporting Summary

Nature Portfolio wishes to improve the reproducibility of the work that we publish. This form provides structure for consistency and transparency in reporting. For further information on Nature Portfolio policies, see our Editorial Policies and the Editorial Policy Checklist.

Statistics

For all statistical analyses, confirm that the following items are present in the figure legend, table legend, main text, or Methods section.

n/a	Confirmed	
☐	☒	The exact sample size (*n*) for each experimental group/condition, given as a discrete number and unit of measurement
☐	☒	A statement on whether measurements were taken from distinct samples or whether the same sample was measured repeatedly
☐	☒	The statistical test(s) used AND whether they are one- or two-sided<br>*Only common tests should be described solely by name; describe more complex techniques in the Methods section.*
☐	☒	A description of all covariates tested
☐	☒	A description of any assumptions or corrections, such as tests of normality and adjustment for multiple comparisons
☐	☒	A full description of the statistical parameters including central tendency (e.g. means) or other basic estimates (e.g. regression coefficient) AND variation (e.g. standard deviation) or associated estimates of uncertainty (e.g. confidence intervals)
☐	☒	For null hypothesis testing, the test statistic (e.g. *F*, *t*, *r*) with confidence intervals, effect sizes, degrees of freedom and *P* value noted<br>*Give P values as exact values whenever suitable.*
☒	☐	For Bayesian analysis, information on the choice of priors and Markov chain Monte Carlo settings
☒	☐	For hierarchical and complex designs, identification of the appropriate level for tests and full reporting of outcomes
☒	☐	Estimates of effect sizes (e.g. Cohen's *d*, Pearson's *r*), indicating how they were calculated

Our web collection on statistics for biologists contains articles on many of the points above.

Software and code

Policy information about availability of computer code

Data collection	*Provide a description of all commercial, open source and custom code used to collect the data in this study, specifying the version used OR state that no software was used.*
Data analysis	*Provide a description of all commercial, open source and custom code used to analyse the data in this study, specifying the version used OR state that no software was used.*

For manuscripts utilizing custom algorithms or software that are central to the research but not yet described in published literature, software must be made available to editors and reviewers. We strongly encourage code deposition in a community repository (e.g. GitHub). See the Nature Portfolio guidelines for submitting code & software for further information.

Data

Policy information about availability of data

All manuscripts must include a data availability statement. This statement should provide the following information, where applicable:

- Accession codes, unique identifiers, or web links for publicly available datasets
- A description of any restrictions on data availability
- For clinical datasets or third party data, please ensure that the statement adheres to our policy

All data generated or analyzed during this study are included in Supplementary Data 1.

Human research participants

Policy information about studies involving human research participants and Sex and Gender in Research.

Reporting on sex and gender	*Use the terms sex (biological attribute) and gender (shaped by social and cultural circumstances) carefully in order to avoid confusing both terms. Indicate if findings apply to only one sex or gender; describe whether sex and gender were considered in study design whether sex and/or gender was determined based on self-reporting or assigned and methods used. Provide in the source data disaggregated sex and gender data where this information has been collected, and consent has been obtained for sharing of individual-level data; provide overall numbers in this Reporting Summary. Please state if this information has not been collected. Report sex- and gender-based analyses where performed, justify reasons for lack of sex- and gender-based analysis.*
Population characteristics	*Describe the covariate-relevant population characteristics of the human research participants (e.g. age, genotypic information, past and current diagnosis and treatment categories). If you filled out the behavioural & social sciences study design questions and have nothing to add here, write "See above."*
Recruitment	*Describe how participants were recruited. Outline any potential self-selection bias or other biases that may be present and how these are likely to impact results.*
Ethics oversight	*Identify the organization(s) that approved the study protocol.*

Note that full information on the approval of the study protocol must also be provided in the manuscript.

Field-specific reporting

Please select the one below that is the best fit for your research. If you are not sure, read the appropriate sections before making your selection.

☒ Life sciences ☐ Behavioural & social sciences ☐ Ecological, evolutionary & environmental sciences

For a reference copy of the document with all sections, see nature.com/documents/nr-reporting-summary-flat.pdf

Life sciences study design

All studies must disclose on these points even when the disclosure is negative.

Sample size	Comparisons between genotypes were made based on n>3 for all experiments and based on mice available for testing. n for each experiment by genotype and sex are provided in Supplementary Data 1.
Data exclusions	No data is excluded.
Replication	Several of the experiments were repeated based on the initial reviewers comments. The following experiments were repeated with a new cohort of animals and the new data is included in this version of the manuscript: 1. western blots, 2. indirect calorimetry, 3. fasting studies, 4. plasma acylcarnitines. The following studies were repeated and additional data added to previous data: 1. palmitate oxidation, 2. echocardiography.
Randomization	No radomization. Comparisons were made between genotypes.
Blinding	Echocardiographer and electrophysiologist who conducted the ERGs were blinded to genotype at the time of testing.

Reporting for specific materials, systems and methods

We require information from authors about some types of materials, experimental systems and methods used in many studies. Here, indicate whether each material, system or method listed is relevant to your study. If you are not sure if a list item applies to your research, read the appropriate section before selecting a response.

Materials & experimental systems

n/a	Involved in the study
☐	☒ Antibodies
☒	☐ Eukaryotic cell lines
☒	☐ Palaeontology and archaeology
☐	☒ Animals and other organisms
☒	☐ Clinical data
☒	☐ Dual use research of concern

Methods

n/a	Involved in the study
☒	☐ ChIP-seq
☒	☐ Flow cytometry
☒	☐ MRI-based neuroimaging

Antibodies

Antibodies used	HADHA, Fisher Scientific, PIPA527348, HADHB, Fisher Scientific, PIPA5117024; ACADVL, Invitrogen, cat# PA529959; Monoclonal: HADHA (3E9B1), Fisher Scientific, 50-173-6347; GAPDH, Santa Cruz Biotechnology, sc-365062 and sc-365062 HRP)
Validation	Statements from the manufacturers website: "PA5-27348 targets HADHA in IF, IHC (P), and WB applications and shows reactivity with Human, Rat and mouse samples. The PA5-27348 immunogen is recombit fragment corresponding to a region within amino acids 459 and 763 of Human HADHA"; "HADHB Polyclonal antibody specifically detects HADHB in Human, Mouse, Rat samples. It is validated for Immunocytochemistry, Immunofluorescence, Western Blot"; "Molecular cell 2018 - Published figure using ACADVL polyclonal antibody (Product # PA5-29959) in Western Blot" (no validation reported on website for monoclonal HADHA); "GAPDH Antibody (G-9) is a mouse monoclonal IgG1 κ, cited in 1,422 publications; Anti-GAPDH Antibody (G-9) is recommended for detection of GAPDH and GAPDH-2 of mouse, rat and human origin by WB, IP, IF, IHC(P) and ELISA"

Animals and other research organisms

Policy information about studies involving animals; ARRIVE guidelines recommended for reporting animal research, and Sex and Gender in Research

Laboratory animals	C57Bl6J is the background of this mouse. Numbers range from 4 to 14 per genotype by sex and are listed in the data file. Most experiments were performed in 10-12 month old mice. When this was not the case, age is given in the text and in the supplementary data file.
Wild animals	na
Reporting on sex	Sex is included in all the figures and reporting.
Field-collected samples	na
Ethics oversight	OHSU IACUC (eIACUC #B11243).

Note that full information on the approval of the study protocol must also be provided in the manuscript.
